# Rheological Behaviour of WMA-Modified Asphalt Binders with Crumb Rubber

**DOI:** 10.3390/polym14194148

**Published:** 2022-10-03

**Authors:** Emilio Turbay, Gilberto Martinez-Arguelles, Tatiana Navarro-Donado, Edgar Sánchez-Cotte, Rodrigo Polo-Mendoza, Elvis Covilla-Valera

**Affiliations:** 1 Department of Civil & Environmental Engineering, Universidad del Norte, Barranquilla 081001, Colombia; 2 Facultad Tecnológica, Universidad Distrital Francisco José de Caldas, Bogotá 111611, Colombia; 3 Faculty of Science, Charles University, 128 00 Prague, Czech Republic

**Keywords:** asphalt binder, crumb rubber, rheology, warm mix asphalt, warm mix technology

## Abstract

Crumb rubber (CR) is one of the materials most widely used in the road infrastructure industry due to its mechanical and environmental benefits as an asphalt binder modifier. Nonetheless, CR decreases the workability of mixes by increasing the viscosity of the binder, leading to an increase in the production temperatures of asphalt mixes. However, warm mix technologies can reduce the temperature demand associated with these processes. The preceding explains the growing interest in producing rubberised asphalt binders incorporating warm mix asphalt (WMA) additives. In this research, the mechanical and rheological properties of a 60/70 penetration grade asphalt binder modified with CR (at a dosage of 15, 18 and 21% by the wet process) and WMA chemical additives (Evotherm M1 and Iterlow T) were investigated. Laboratory tests included penetration, softening point, rotational viscosity, frequency sweep through dynamic shear rheometer (DSR), and multiple stress creep recovery (MSCR) tests. The results indicate that CR increases the stiffness of the asphalt binder, which is reflected in a lower penetration grade and improved softening point. It also improves its rutting resistance but decreases fatigue performance. Furthermore, it has been shown that under the conditions studied, the higher the CR content, the more elevated the degree of stiffness and performance of the asphalt binder. On the other hand, WMA technology decreases asphalt stiffness and performance at high temperatures.

## 1. Introduction

The compromising practices concerning the management and disposal of tires are a problem worldwide, and Colombia is no exception. The burning of these tires generates high levels of air pollution since their combustion generates the emission of pollutants such as particles, carbon monoxide (CO), sulphur dioxide (SOx), nitrogen oxides (NOx), and compounds volatile organics (VOCs) [1,2,3]. Therefore, the disposal of tires has become a global problem due to their durability and the large volume they generate. Likewise, the disposal of these wastes represents potential dangers to human health in some circumstances and a significant risk to the environment [4,5,6]. In Bogotá (Colombia), the Urban Development Institute (IDU) mentions that 18,861 tons of tires are generated per year, of which the majority is used for energy purposes (71.9%), another considerable amount is re-treaded (17.2%), and the rest is used for crafts and other purposes (8.5%) [7]. Nowadays, crumb rubber (CR) is widely used as an asphalt binder additive in Colombia [8] because these rubberised binders improve pavement performance, represent an increase in service life, and decrease maintenance costs [9,10,11,12]. CR tends to provide asphalt mixtures with similar benefits as SBS and SBR polymers, but at higher doses and with the advantage that it has a positive impact on the environment as described above but is sensitive to decomposition and the absorption of oxygen [11,13]. As is well known, the addition of CR in asphalt binders implies an increase in the viscosity of the asphalt binders, which translates into higher production temperatures, generating a negative impact on the environment [9,14,15].

Given the need to reduce the industrial carbon footprint, new techniques are being developed in road infrastructure. For instance, warm mix asphalt (WMA) technology aims to significantly reduce the mixing, laying and compaction temperature of the mixture to reduce the emissions of harmful gases [16,17,18]. The literature identifies three main techniques for the addition of WMA: foaming processes, organic additives and chemical additives [2,19,20]. Due to the functionality of these new technologies, these additives can be considered “green” tools since the WMA replaces the hot mix asphalt (HMA), having a positive influence on global warming, air pollution, and the efficiency of the fuel; and thus decreasing the carbon footprint [21,22,23,24]. The use of WMA has increased due to many benefits that this technology can offer, such as: greater comfort and safety for construction workers, better workability at low temperatures, greater haulage distances, rapid rotation for traffic, extended paving window, lower power consumption and enabling the implementation of higher reclaimed asphalt pavement (RAP) content [19,25,26,27,28]. Furthermore, the WMA technology with CR is consistent with the idea of sustainable development, which focuses on the environment and economic development, social development, and environmental protection [26,29,30]. In the literature, it has been found that binders modified with WMA and CR (WMA-CR) positively impact the environment and the construction industry; however, the results cannot be generalised and need to be further explored [31,32,33]. For this reason, this research intends to evaluate the short-term behaviour of WMA modified with CR to investigate the potential benefits of the durability and short-term performance of Colombian modified asphalt binders.

The structure of this article is as follows. Section 2 presents a literature review on the performance of binders and asphalt mixtures modified with CR and WMA. Section 3 shows the experimental design (description of the methodology and tests performed) and materials used in the investigation. Subsequently, Section 4 exhibits the results, discussion of the tests, and the statistical analysis of the parameters analysed. Finally, Section 5 gives the main conclusions and recommendations of this research.

## 2. Background

This study focuses on the rheological behaviour of an asphalt binder modified with WMA chemical additives and CR. A brief review of this type of modified asphalts was carried out. The benefits and limitations in terms of performance and the influence of ageing on the properties of asphalt binders were also considered.

### 2.1. Warm Mix Technology

WMA technology emerged in response to environmental regulations, which became more stringent between 2000 and 2015 [34,35,36]. In order to reduce the amounts of CO_2_ produced and the energy consumed, several technologies were developed based on sustainable development, of which WMA stands out [34,35,36,37]; studies show that HMA, in the presence of this technology, has a similar performance [38,39,40]. Previous studies show that WMA decreases resistance to moisture damage, presenting a higher percentage of adhesion failures than HMA [10,41]. Likewise, the addition of SBS and WMA significantly increases the stiffness and rutting resistance of the asphalt binder [40,42]. These additives also generate a potential increase in resistance to fatigue and temperature cracking [43,44,45]. Other research concluded that the addition of Sasobit decreased the viscosity of the asphalt binder and improved the rheological performance at high temperatures and low-temperature cracking of the aged binder [46]. In contrast, the addition of Evotherm did not significantly affect the performance of the base binder and could prevent the increase in stiffness of polymer-modified asphalts [47].

### 2.2. Crum Rubber (CR)

Tires are mainly composed of vulcanised rubber, some polymers, and various reinforcing materials, mainly made of natural rubber, which is very strong and durable [5,15]. Polymers are materials composed of high-molecular-weight macromolecules joined by covalent bonds; these materials have been widely used in the road infrastructure to modify asphalt layers [20,48,49]. One of the polymers that stands out the most in this field is CR [50,51]. CR as an additive for asphalt pavement has been used for more than 50 years [52]. Researchers point out that the addition of CR in asphalt binders affects their rheological performance [53,54,55].

One investigation noted that the addition of inorganic additives improved the rutting resistance of CR-modified asphalt binders [56]. In contrast, another research found that adding wax to CR binders significantly improves rutting resistance and its resistance to ageing [33,57]. It also found that CR with waxes presents good performance to rutting and fatigue resistance [10,20], but ageing induces cracking at low temperatures [27]. Furthermore, it was found that mixtures with CR and Evotherm exhibited better resistance to rutting, fatigue and moisture damage [58]. Another researcher analysed the effect of Sasobit and Evotherm on a CR-modified asphalt binder and found that Sasobit had a positive effect at high temperatures and Evotherm had a slightly negative effect but showed better fatigue behaviour [59].

On the other hand, other researchers studied CR with wax-based additives through more specialised tests such as MSCR and LAS and found that these additives improve rutting and fatigue resistance [13,60]. The CR mixture combined with WMA additives is expected to have a promising long-term performance in terms of long-term fatigue [27]. Another study pointed out that the chemical additives’ binders showed higher resistance to fatigue than those modified with organic additives and the control mixtures [61]. Other researchers suggest that countries with warm climates are ideal for WMA-CR-modified binders [62]. WMA alters the properties of the microstructure of the binder, which can be related to the increase in the surface roughness of the pavement [63].

## 3. Materials and Experimental Design

### 3.1. Materials

#### 3.1.1. Asphalt Binder

The base asphalt binder used in this research has a penetration grade of 60/70 dmm, commonly used for warm climates and widely used in Colombia, provided by a representative Colombian national supplier. Table 1 shows the characteristics of the material.

#### 3.1.2. Crumb Rubber (CR)

This study used three CR contents of 15, 18 and 21% of 30 mesh with a maximum size of 0.6 mm. These contents were selected due to the suggestions and limits allowed by the Colombian standards for modified asphalt binders [64,65]. Likewise, scanning electron microscopy (SEM) images were also analysed to identify the elemental chemical composition and morphology of the CR particles.

**Figure 1:**
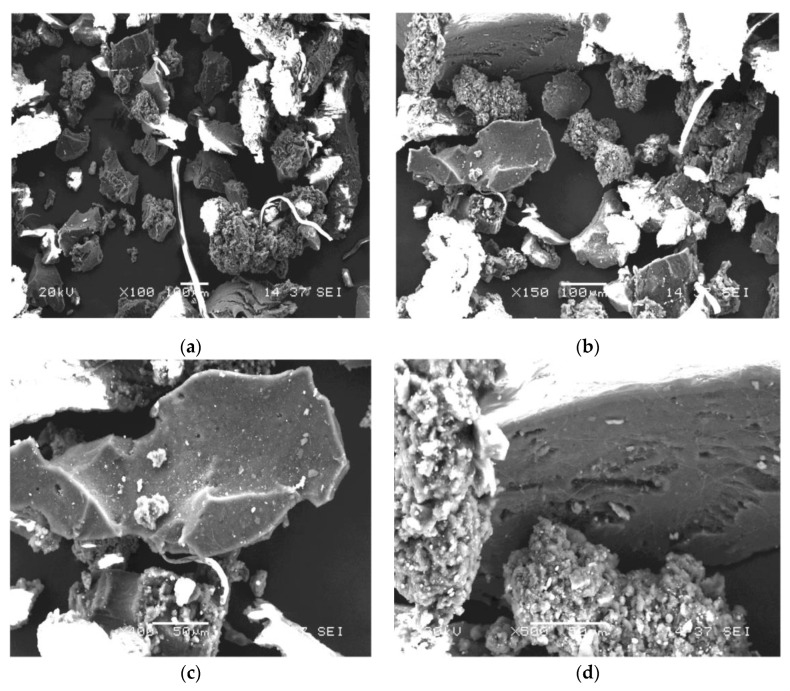
SEM analysis of CR: (**a**) ×100; (**b**) ×150; (**c**) ×400; (**d**) ×600.

Figure 1 of the SEM analysis shows the different particle sizes through the different scales studied (seen in the gradation). The rubber particles have an irregular shape and a spongy appearance [66]. It was reported that a large surface area would promote the interaction of asphalt and rubber, causing more rapid absorption of the light components of the asphalt into the rubber, which will improve the properties of the final binders [67]. On the other hand, small impurities were also found in this material.

#### 3.1.3. WMA Additives

**Figure 2:**
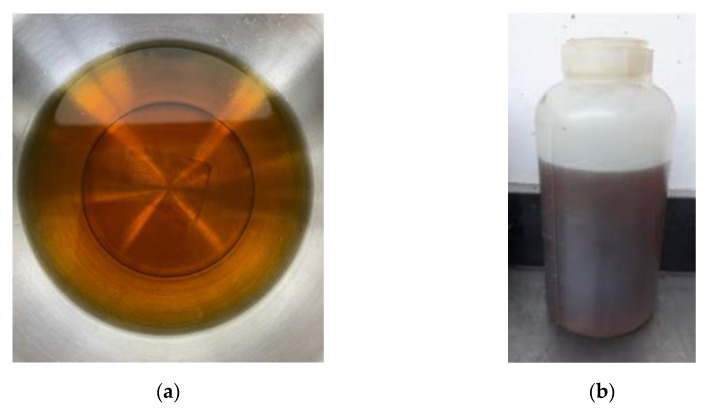
WMA additives: (**a**) Evotherm M1; (**b**) Iterlow T.

The purpose of the WMA chemical additives is to reduce the internal friction within the mixes at low temperatures without influencing the viscosity of the asphalt binders; this is achieved because the mixing temperature accelerates the emulsion breaking and the evaporation of water, and later, the binder coats the aggregates [32,58]. In this research, Evotherm M1 and Iterlow T (Figure 2) were used at a dose of 0.3% by weight of the asphalt binder. Table 2 shows some of the properties of these additives.

#### 3.1.4. Production of Rubberised WMA Binders

**Figure 3:**
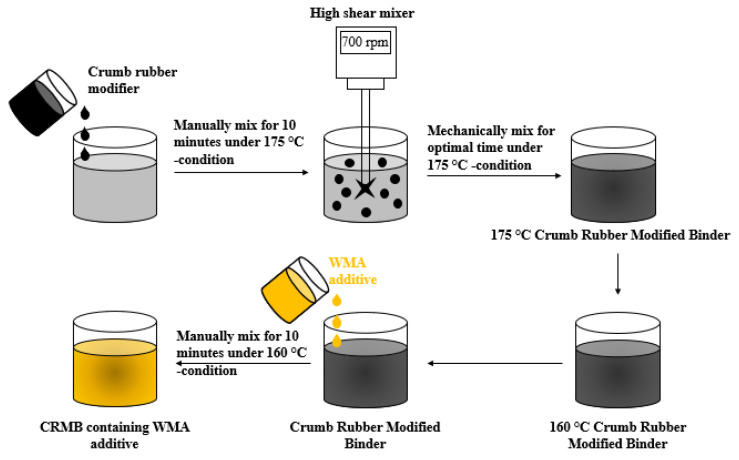
Mixing procedure for preparing CR-modified binders in this research.

The production of the modified asphalt binders was carried out by employing the wet process. The literature review defined specific parameters to establish a mixing procedure. The literature shows a wide range of mixing speeds ranging from 200 rpm [68] to 8000 rpm [29,39], and the most commonly used mixing rate for CR-modified binders is 700 rpm [29]. These mixing temperatures (< 190 °C) do not make a significant difference; this is mainly because, at temperatures above this, the CR particles depolymerise and age, stiffening the asphalt binder. The crumb rubber percentages evaluated in this study were 15, 18, and 21%, and mixing times were 30, 45, and 60 min. The modified asphalt mixing procedure was carried out at a temperature of 175 °C. First, the asphalt is heated to the selected temperature. Then, the crumb rubber is mixed manually during 10 min, followed by the mechanical mixing procedure with a high shear mixer at 700 rpm during optimum mixing time. The WMA additive is added for 10 min at a temperature of 160 °C. The mixing procedure is illustrated in Figure 3.

### 3.2. Testing Procedure

**Figure 4:**
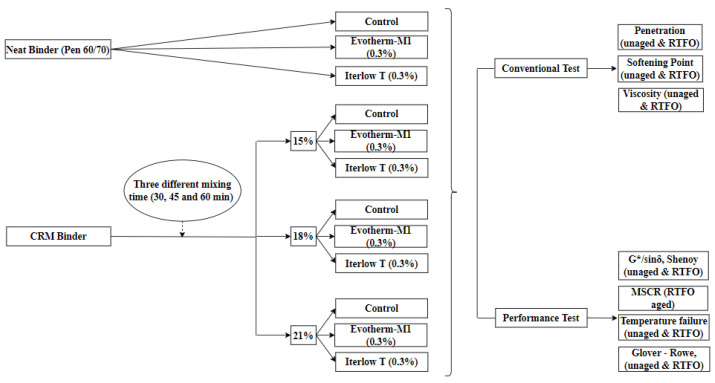
Experimental design used in this research.

The experimental program of this study is shown in Figure 4. In this section, the performances of the modified and unmodified binders will be evaluated through the laboratory tests described later. Tests were performed on samples in original and RTFO residue state test.

### 3.3. Conventional Tests

The conventional tests were rotational viscosity (RV), degree of penetration and softening point. With these tests, the stiffness and behaviour of the binders at high service temperatures were evaluated. The standards were as follows: ASTM D4402, ASTM D5 and ASTM D36.

### 3.4. Frequency Sweeps

For the high-temperature performance of the binder, frequency sweeps (ASTM D7175) were performed through DSR with the 25 mm diameter plates from 0.1 to 100 rad/s frequency at different temperatures (46, 52, 58, 64, 70, and 76 °C), all measurements were performed at a strain level of 12% (for both unaged and RTFO aged samples). The objective of this test is to analyse the parameters complex shear modulus (G*) and phase angle (*δ*), which describe the viscoelastic behaviour of the material [69,70,71]. Taking into account the principle of time-temperature superposition, the viscoelastic response of each of the asphalt binders can be analysed through the Black diagram, which plots G* vs. *δ* [29,68]. In addition, through the Cole–Cole space, the behaviour of the imaginary part (loss modulus or viscous portion) and a real part (storage modulus or elastic portion) can be observed, that is, the asphalt in the complex plane [29,72]. The G*/sin *δ* parameter evaluates the rutting resistance, and the higher the parameter indicates a higher rutting resistance. Furthermore, the Superpave rutting parameter, according to research, does not show a good correlation with the high-temperature performance of some binders, especially polymer-modified ones [73,74]; therefore, the new parameter suggested by Shenoy (Equations (1) and (2)) [75] is employed:

(1) $$G^{*}\times {\left(\sin\delta \right)}^{9}\to \delta <55{}^{\circ},$$

(2) $$\frac{G^{*}}{1-\left(\frac{1}{\tan\delta \times \sin\delta }\right)}\to \delta >55{}^{\circ},$$

where G* is the complex modulus, and *δ* is the phase angle.

Likewise, the Glover–Rowe parameter allows to analyse and understand the fatigue behaviour of the asphalt binder [71,76,77], which is calculated by the following equation:

(3) $$G-R=G^{*}\;\left(\frac{\cos\delta^{2}}{\sin\delta }\right).$$

### 3.5. Multiple Stress Creep Recovery (MSCR) Tests

The MSCR test (ATM D7405) evaluates the resistance to rutting of a sample aged by RTFO (ASTM D2872) of an asphalt binder through the DSR at 64 °C. The parameters produced by this test are: non-recoverable creep compliance (*J_nr_*) (Equation (4)) and recovery percentage (*R*) (Equation (5)), the first is determined by dividing the non-recoverable shear stress by the applied shear stress, while the R analyses the recovery of the material after receiving a load [78,79].

(4) $$J_{nr}\left(\sigma ,N\right)=\frac{\varepsilon_{r}-\varepsilon_{0}}{\sigma },$$

(5) $$R\left(\sigma ,N\right)=\frac{\varepsilon_{c}-\varepsilon_{r}}{\varepsilon_{c}-\varepsilon_{0}},$$

where σ is the applied stress (kPa); *N* is the number of creep and recovery cycles; $J_{nr}\left(\sigma ,N\right)$ is the measured non-recoverable creep compliance at a given cycle (kPa^−1^); and R(σ,N) is the measured recovery at a given cycle (%).

## 4. Results and Discussion

### 4.1. Selection of Optimum Mixing Time

In order to determine the optimum mixing time for each of the CR contents (15, 18 and 21%), three mixing times (30, 45 and 60 min) were evaluated. For each sample, a frequency sweep (46–76 °C) by DSR was adopted.

**Figure 5:**
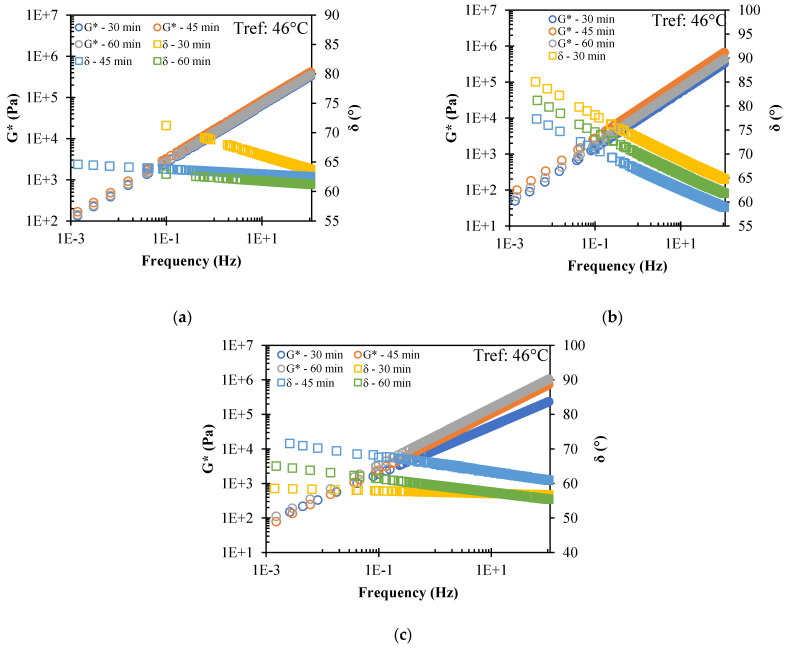
Master curve of G* and *δ* of different mixing times at 46 °C of: (**a**) 15% CR; (**b**) 18% CR; (**c**) 21% CR.

Figure 5 shows the master curves for both G* and *δ*. For 15% CR, it can be observed that for G*, there is no significant difference between the mixing times, while for *δ*, it increases as the mixing time increases; therefore, the optimum mixing time for 15% CR is 45 min. Meanwhile, for the 18% content of CR, a better rutting performance (higher G*/sin *δ*) was obtained for the mixing time of 45 min, which implies a higher G* and a lower *δ*; therefore, the mixing time of 45 min was selected as optimal for the 18% CR content. Likewise, for the 21% CR, the frequency sweep showed a trend that as the mixing time increases, the higher the G*, the mixing time of 60 min also presented a higher G*; it is therefore concluded that the optimum mixing time for the 21% CR content is 60 min. Table 3 summarises the optimum mixing times for each CR content.

### 4.2. Conventional Tests

It is worth mentioning that for the conventional tests, six samples were obtained for each one, and the average of these was used for the analysis. In addition, these tests followed ASTM D6114 standards for asphalt–rubber binders.

**Figure 6:**
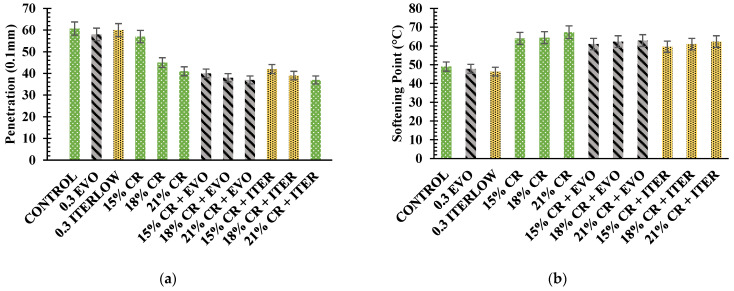
Results of conventional test: (**a**) penetration; (**b**) softening point.

The penetration test results (Figure 6a) indicated that adding CR decreases the penetration grade as the CR content increases. Therefore, it can be concluded that as the CR content increases, the asphalt binder presents a higher stiffness (lower penetration grade). This may be due to the crumb rubber components; modifying the asphalt could increase the asphaltenes/resins ratio. The literature establishes that adding crumb rubber implies decreased aromatics and saturated content [11,80]. The addition of Evotherm M1 reduced the penetration grade by 4.5% and Iterlow T by 1.15% compared to the control sample. At the same time, the addition of CR reduced the penetration grade by 29.8, 25.9 and 32.5% for CR contents of 15, 18 and 21%, respectively. Therefore, the addition of Evotherm M1 in the CR-modified binder reduction of 29.8, 15.6, and 9.8% for the CR contents of 15, 18, and 21%, respectively. Likewise, adding Iterlow T meant a reduction for the CR-modified binders of 26.3, 13.3, and 7.3% for the contents mentioned above.

The softening point or ring and ball test (Figure 6b) show that the addition of CR in the asphalt increases the softening point; therefore, as the CR content increases, the softening point increases because the CR produces changes in the elastic components, which is reflected in better resistance to temperature changes. Likewise, the addition of WMA chemical additives decreases the softening point in both unmodified and modified asphalt, with Iterlow T having a more pronounced effect than Evotherm M1, which may be due to the chemical additives’ components or low boiling and melting points. The addition of Evotherm M1 reduced the softening point by 2.5%, while Iterlow T reduced it by 5.5% compared to the control sample. In contrast, adding CR increased the softening point by 30.6, 31.4, and 37.4% for CR contents of 15, 18, and 21%, respectively. The addition of Evotherm M1 in CR-modified binders accounted for a 4.7, 3.3, and 6.6% reduction for CR contents of 15, 18, and 21%, respectively. On the other hand, Iterlow T reduced the CR contents studied by 6.9, 5.3, and 7.4%.

**Figure 7:**
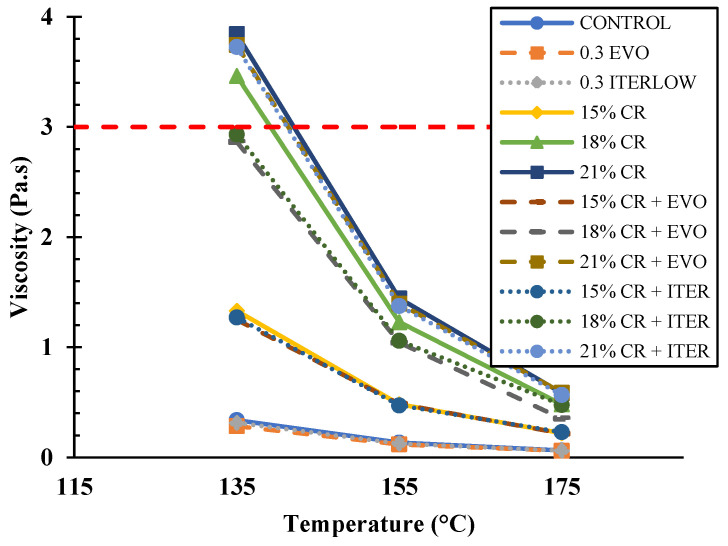
Results of rotational viscosity (RV) test.

On the other hand, the rotational viscosity test (Figure 7) allows for analysis of the workability of the asphalt and determining its mixing and compaction temperatures. In this study, 135, 160, and 175 °C were considered because construction temperatures for unmodified asphalts are generally below 135 °C, but these temperatures are much higher for modified asphalts. From the literature, it can be noted that crumb rubber increases resins and asphaltenes, thus decreasing the colloidal stability index and increasing viscosity [11,80]. As expected, the addition of CR increased the viscosity by 249.2, 663.5, and 82.6% for CR contents of 15, 18, and 21%, respectively, at a temperature of 175 °C compared to the control sample. On the other hand, Evotherm M1 and Iterlow T slightly reduced the viscosity of CR-modified binders at all temperatures but were still superior to the control. In the literature, the effect of WMA chemical actives is discussed because it should not alter the viscosity since its purpose is to reduce the friction between aggregates and asphalt [58], but in other research, it is observed that the additive does slightly reduce the viscosity [42,81], which is also observed in this research.

### 4.3. Frequency Sweep

**Figure 8:**
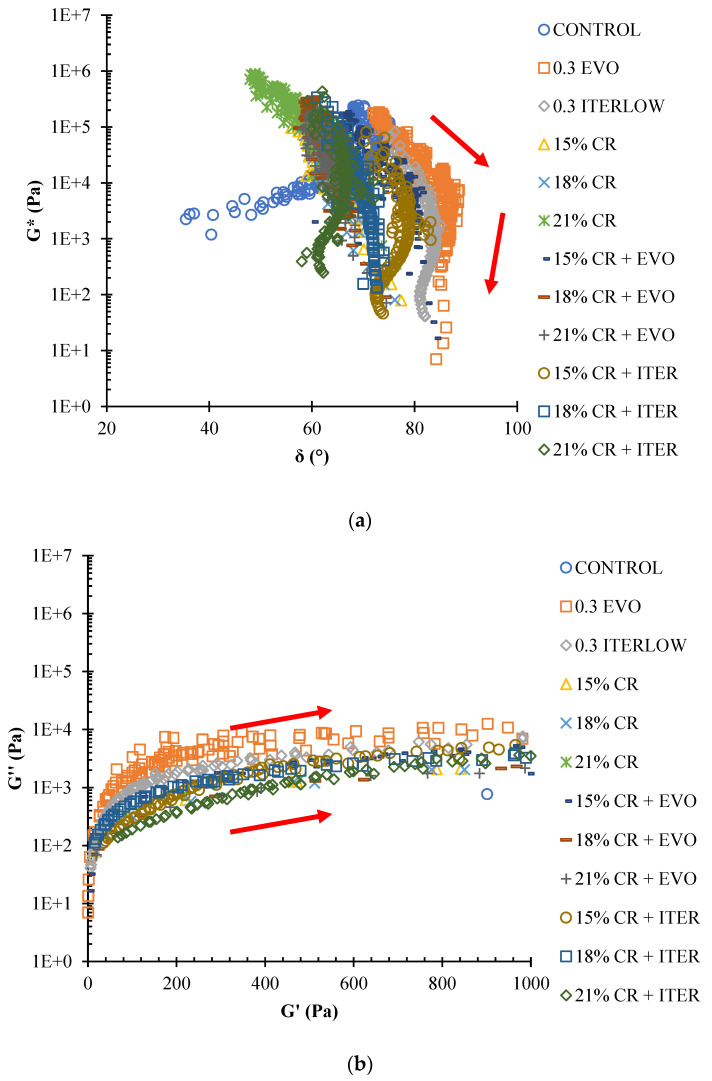
(**a**) Black diagram; (**b**) Cole–Cole space.

The information obtained from the frequency sweeps was analysed to describe the rheological behaviour of the modified and unmodified asphalt binders. As mentioned above, the Black diagram and Cole–Cole space. In the Black diagram (Figure 8a), it is observed that all the materials are thermorheologically simple due to the continuity of all their data; on the other hand, it is also observed that all the modified asphalts present a similar viscoelastic response among them. From the Black diagram, it can also be observed that the CR-modified asphalt binders show an inverse “S” as in previous investigations [20,29,82], only the control asphalt and the one modified only with Evotherm M1 and Iterlow T present a slightly different response.

In the Cole–Cole space (Figure 8b), in all samples, the elastic component is greater than the viscous component, except in the original asphalt, the one modified with Evotherm M1 and the Iterlow T is reflected in a high phase angle. The addition of CR confirmed an increase in the elastic component as expected since CR improves the elastic properties of asphalt, and the higher the CR content, the more the elastic component increases; this is because the swelling and dispersion processes of the crumb rubber give the asphalt its elasticity [11]. In addition, the WMA chemical additives in CR-modified binders decrease the elastic component but still show the same trend and remain superior to the original asphalt binder.

**Figure 9:**
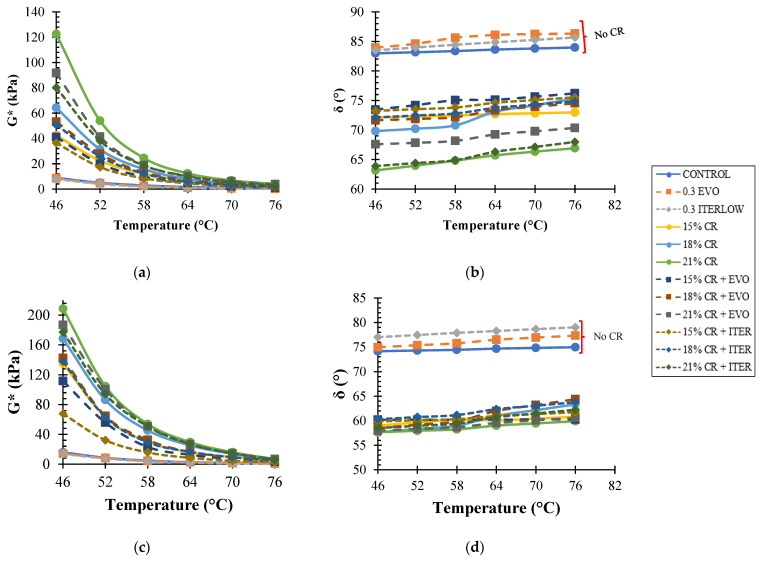
(**a**) G* vs. temperature for unaged sample, (**b**) *δ* vs. temperature for unaged sample, (**c**) G* vs. temperature for RTFO aged sample, (**d**) *δ* vs. temperature for RTFO aged sample.

Likewise, to observe more clarity in the behaviour of the rheological parameters, G* and *δ* were plotted versus temperature independently of the unaged and RTFO-aged samples. In Figure 9, it can be concluded that as the temperature increases, G* decreases. In addition, the samples modified only with Evotherm M1 and with Iterlow T had a lower G* than the control sample at all temperatures. On the other hand, all CR-modified binders had a higher G* than the control sample; at 64 °C, there was an increase in G* of 304.5, 403.2, and 683.4% for CR contents of 15, 18, and 21%, respectively, compared to the control sample; the addition of CR showed a trend directly proportional to the increase in G* at all temperatures analysed. WMA chemical additives in CR-modified binders decreased G* for all contents, but all presented a higher G* than the control sample at all temperatures. The addition of Evotherm M1 decreased the G* of the control sample by 34.4%, while for the CR-modified binders, by 23.5, 10.6, and 16.9% for the 15, 18, and 21% contents. Likewise, Iterlow T reduced G* by 37.6%, and for CR-modified binders, it represented a reduction of 29.1, 26.8, and 19.8% for the respective contents. Finally, given the results described above, it can be concluded that the chemical additives decrease the effect of the CR, the Iterlow T more than the Evotherm M1, but present a higher complex modulus than the control.

RTFO aged samples show a higher G*, this is due to the fact that ageing induces some of the less polar fractions to change to more polar fractions, causing the molecular weight to increase, resulting in lower ductility and higher viscosity, which implies a higher stiffness [11]. The G* of the control sample had a 60.5% increase in G* compared to the unaged sample, while the CR-modified binders had a more notable increase for all their contents (146.9, 217.7, and 183.8% increases). Likewise, WMA-CR-modified binders showed a lower G* than CR-modified binders without WMA but a higher G* than the control sample. On the other hand, the phase angle of these samples aged by RTFO showed a decrease in that parameter; unlike the control samples, the Evotherm M1 presented lower phase angles than the Iterlow T modified ones.

**Figure 10:**
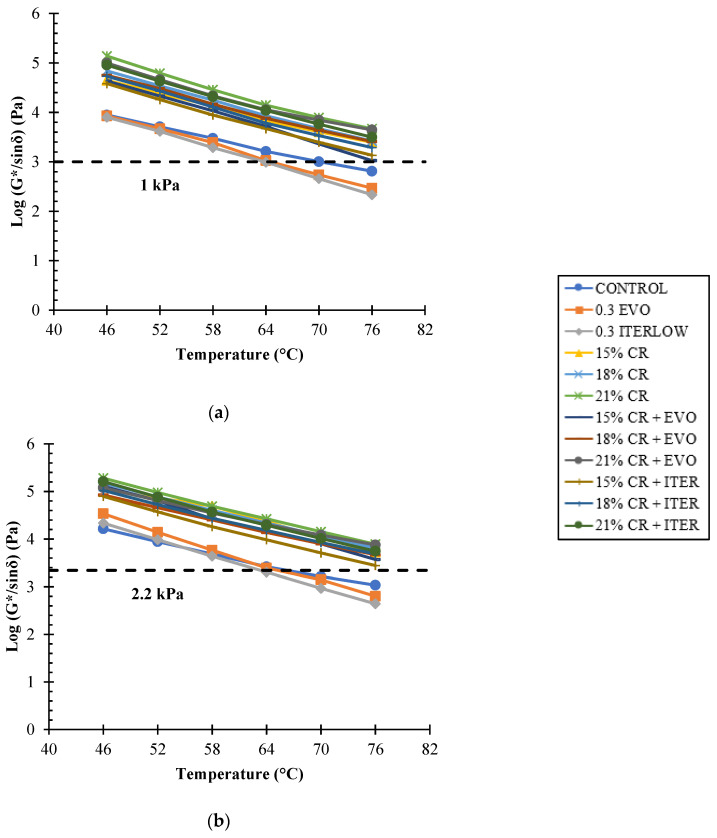
Log (G*/sin *δ*) vs. temperature for: (**a**) unaged; (**b**) RTFO aged samples.

With the data obtained, the failure temperatures were calculated for each sample. This procedure was carried out by calculating the temperatures at which the rutting parameter (G*/sin *δ*) is equal to 1 kPa for the unaged samples and 2.2 kPa for the samples aged with RTFO. The preceding can be observed in Figure 10.

**Figure 11:**
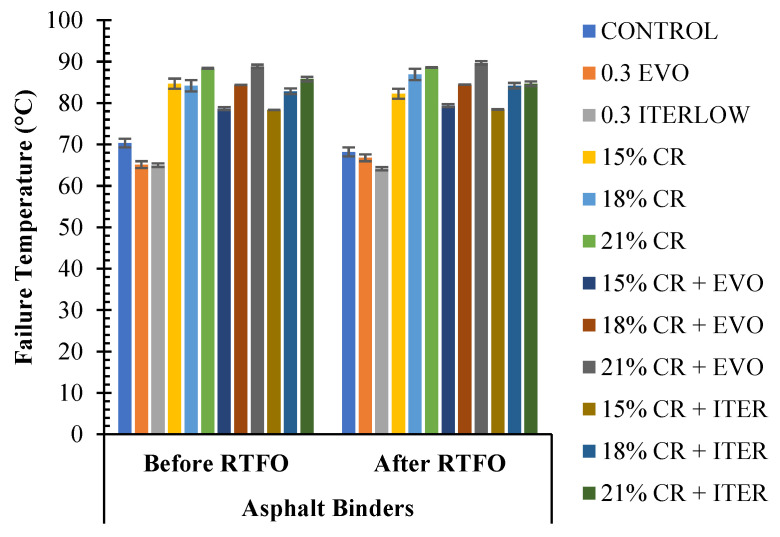
Calculated failure temperatures for the modified and unmodified samples.

To complement the above information, Figure 11 shows the failure temperature for each sample. It can be concluded that the addition of CR significantly improves the high-temperature performance of asphalt binders, as well as their sensitivity to these temperatures, because this additive improves the stability of the asphalt due to its crystalline network/lattice structure [26,40,83], the addition of WMA additives led, in some cases, to a decrease in PG. However, the performance is still far superior to that of unmodified asphalt binders [33].

**Figure 12:**
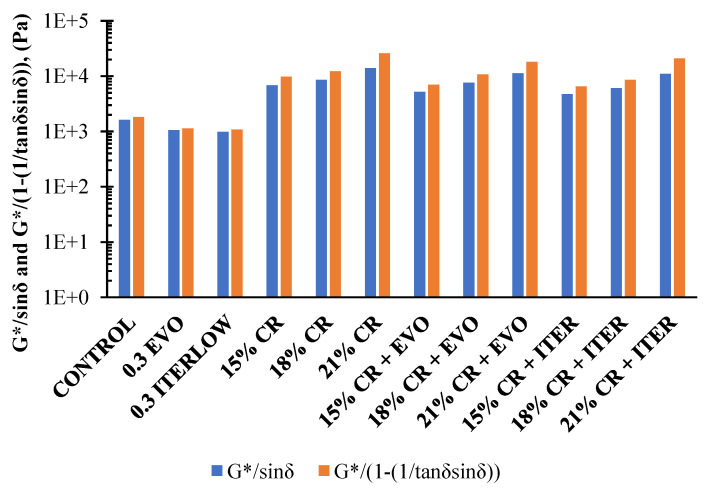
Comparison between the Shenoy and Superpave parameters at 64 °C in the unaged samples.

Continuing the analysis of the rutting behaviour of the asphalt binders, the Shenoy parameter is then calculated. It can be mentioned that this parameter is very similar to that of the Superpave protocol; the effect of the additives used in this research maintains the same trends in the results of this parameter, in addition to the fact that the effect of ageing is also observed in the RTFO aged samples. The most notable difference between the Shenoy and Superpave parameters is that slightly higher values are obtained in the former. Figure 12 presents the comparison of the Shenoy and Superpave parameters for the unaged samples.

Table 4 shows the sensitivity of the parameters analysed for each CR content and the WMA, Evotherm M1, and Iterlow T additives. It can be concluded that for CR contents from 0 to 18% and from 0 to 21%, the changes in the G*/sin *δ* parameter are less pronounced compared to G*/(1 − (1/(tan *δ* * sin *δ*)), this indicates that the Shenoy parameter is more sensitive to the changes in asphalt structures generated by the modifying agents evaluated. As a result, a more accurate description of the non-recoverable strains is achieved [48,79].

**Figure 13:**
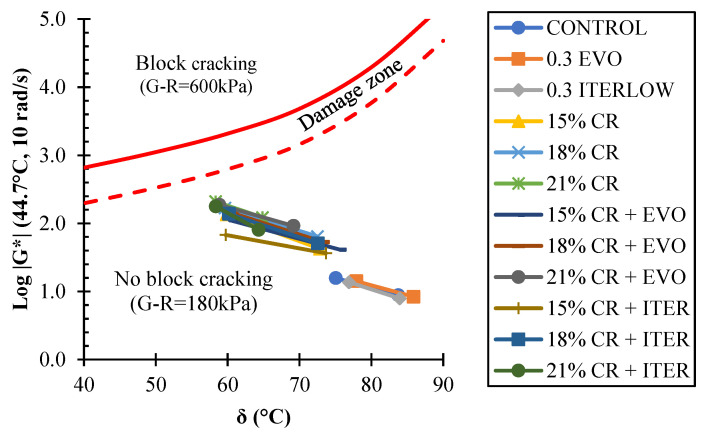
Black diagram for G–R parameter.

On the other hand, the Glover–Rowe (G–R) parameter allows us to understand the fatigue performance of asphalt binders. The analysis was performed for unaged and RTFO aged samples in this research. G–R parameter was calculated through a frequency sweep in the DSR at a temperature of 44.7 °C, and a frequency of 10 rad/s due to these conditions is equivalent to that of 15 °C and 0.005 rad/s [84,85]. Figure 13 shows the results obtained for the G–R parameter. It can be mentioned that the addition of CR meant an increase in the G–R parameter, which translates into a decrease in fatigue resistance. On the other hand, the WMA chemical additives improved the performance of these binders, even in the presence of CR, the Iterlow T having more noticeable benefits than the Evotherm M1.

### 4.4. Multiple Stress Creep Recovery (MSCR) Tests

**Figure 14:**
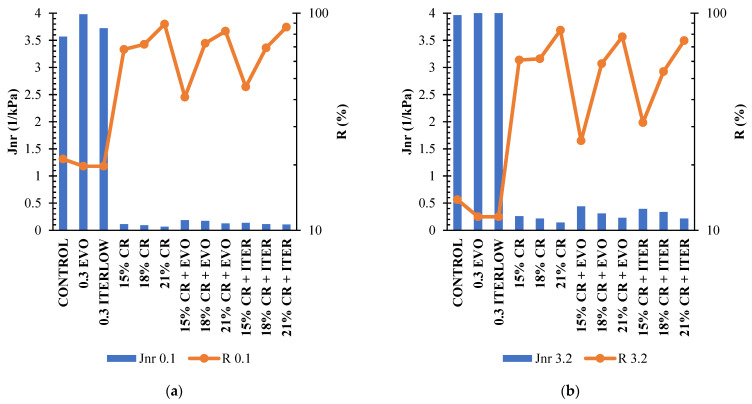
*J_nr_* and R of asphalt binders (**a**) under 0.1 kPa load and (**b**) under 3.2 kPa load.

From Figure 14, it was observed that the addition of CR significantly increased rutting resistance; the addition of Evotherm M1 and Iterlow T to CR-modified binders was reflected in a decrease in rutting resistance but still performed much better than the control sample. The asphalt binder modified only with Evotherm M1 had an increase of 2.4% of *J_nr_* 3.2 and the Iterlow T an increase of 4.5% compared to the control sample; also, the decrease in *J_nr_* 3.2 of 93.4, 94.6, and 96.4% were observed for the contents of 15, 18 and 21% CR, respectively. Furthermore, an increase in *J_nr_* of CR-modified binders with Evotherm M1 of 68.8, 43.5, and 60.8% was presented for 15, 18, and 21%, respectively, compared to those modified only with CR. At the same time, the addition of Iterlow T represented a decrease of 51.13, 56.48, and 51.75% for CR-modified binders. On the other hand, the *J_nr_* results are congruent with those obtained with frequency sweeps, showing increased asphalt stiffness and better G*/sin *δ* parameter behaviour (high G* and low *δ*). With the addition of CR and a slight decrease in rutting resistance of CR-modified binders with WMA compared to those modified only with CR. Although the addition of Evotherm M1 and Iterlow T to the CR-modified binders increased the *J_nr_* parameter, these modified asphalts still showed higher rutting resistance than the control sample.

Likewise, the percentage recovery is used to determine the elastic, and the stress-dependent response of the asphalt binders analysed [27,60]. The percentage recovery results mirrored those obtained with *J_nr_*, i.e., the CR-modified samples showed a considerable increase compared to the control sample. This increase in R goes hand in hand with the increase in CR content. The CR-modified binders with Evotherm M1, and Iterlow T showed the same trend but with slightly lower percentage values, presenting Evotherm M1 decreases of 57.6, 5.0 and 6.8%, for contents of 15, 18 and 21%, respectively, and Iterlow T decreases of 48.5, 12.7 and 10.5%. The results obtained for these parameters were as expected since the CR improves the elastic response compared to the original asphalt, which these results can confirm.

On the other hand, research has shown that the *J_nrdiff_* parameter does not best characterise the stress sensitivity of modified asphalts [27,86]. Moreover, they recommend the alternative parameter *J_nrslope_* defined by the following equation (Equation (4)):

(6) $$Jnrslope=\frac{Jnr_{3.2}-Jnr_{0.1}}{\tau_{3.2}-\tau_{3.2}},$$

where *J_nr_* is creep compliance, and *τ* is applied stress. Table 5 summarises all the parameters obtained from the MSCR test for the modified and unmodified asphalt binders. Most of the *J_nrdiff_* exceed the 75% limit; for this reason, the *J_nrslope_* parameter described above is considered. The lower the *J_nrdiff_* value, the lower the binder sensitivity to stress change [86]. It is observed that the addition of CR increases the binder sensitivity to such stress changes. In contrast, the addition of Evotherm M1 and Iterlow T slightly decreases the binder sensitivity.

**Figure 15:**
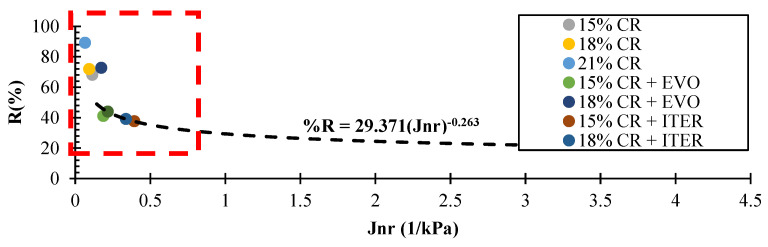
Plot of percentage recovery vs *J_nr_* for modified asphalt binders.

Figure 15 shows the graph of *J_nr_* vs. R, in which we can observe which samples have a higher performance and resistance to rutting since the objective is to have a lower *J_nr_* and a higher R. The data is sought to be in the box bordered in red, which is the case in this case study, i.e., the CR-modified binder samples with and without WMA show a considerable increase in rolling resistance according to the data obtained from the MSCR test. On the other hand, the representative line of R (%) = 29.371 (*J_nr_*) − 0.263, proposed by AASHTO M332, can also be observed in this graph, which establishes that points above this line present an acceptable amount of elastomeric polymers in the asphalt binder. Under these conditions, the sample that does not meet the acceptable level is the asphalt modified with 15% CR and Evotherm M1, which indicates that most of the samples with the different modifiers analysed in this study meet the sufficient amount for each modifier.

### 4.5. Statical Analysis

A comparison of the averages was carried out through an analysis of the variance of the data (ANOVA). This analysis was performed using R Studio software, in which a *p* < 0.05 signified a significant change. Table 6 summarises the significance of the results obtained from the rheology tests. From the viscosity at 135 °C, it can be concluded that only for the 18% content did the addition of both WMA additives represent a significant change, in addition to the fact that the addition of CR was significant for all contents in comparison with the control samples, even this trend is observed in the presence of the WMA additives. For viscosity at 175 °C, only the 18% CR sample modified with Evotherm M1 significantly decreased compared to the CR-modified binder. As for the 135 °C viscosity, the presence of CR with and without WMA represented a significant increase. Likewise, for the Superpave protocol rutting resistance parameter (G*/sin *δ*), the addition of Evotherm M1 and Iterlow T indicated a significant decrease in that parameter for all control samples. The increase in rheological performance due to the modifying agents in asphalt is because CR is composed of several materials, including many polymers, which increase the elastic component of the asphalt, increasing deformation resistance, i.e., asphalt binders modified with RC contain more heavy fractions, increasing G* and resulting in a lower phase angle. It should also be mentioned that the heating of the asphalt can influence the stiffening of this material.

Overall, CR addition significantly increased the rutting parameter. For *J_nr_*, the addition of only Evotherm M1 for the control sample and the addition of Iterlow for the 15% CR sample did not represent a significant increase in the parameter; for the others, the WMA implied a significant increase. Furthermore, the addition of CR did indicate a significant decrease. Finally, in the G–R parameter, the addition of the WMA chemical additives did not represent a significant increase or decrease compared to the CR-modified binder samples. The addition of CR showed a significant increase with and without the addition of WMA.

## 5. Conclusions and Recommendations

This research studied the short-term rheological behaviour (unaged and RTFO aged samples) of a 60/70 dmm asphalt binder. The modifying agents used were CR at 15, 18, and 21%, in addition to two chemical WMA additives: Evotherm M1 and Iterlow T. This study presents the following conclusions:

- It can be mentioned that more mixing time is required as the CR content increases if the temperature is kept constant. In addition, the viscosity should not be the most crucial parameter for understanding the mixing process for this type of modification.
- It was observed from the conventional tests, penetration, and softening point that CR increases the stiffness of the asphalt binder, and WMA tends to decrease this resistance slightly. This may be due to the additive components influencing the chemical properties of the asphalt, or during the mixing process, the asphalt binder was stiffened by heating.
- As for the viscosity, the results show that as the CR increases, the viscosity increases. WMA additives tend to decrease viscosity slightly. Mixing temperatures below 165 °C are recommended for these modified asphalt binders.
- It is concluded that as the CR content increases, the rheological performance improves due to higher values of the Superpave parameter obtained for the resistance to rutting in the frequency sweeps. On the other hand, WMA additives slightly decreased this resistance. Likewise, the Shenoy parameter was also analysed, which presented a behaviour very similar to the Superpave parameter but with slightly higher values. This parameter showed greater sensitivity to the contents of the additives compared to the fact that to the Superpave parameter.
- Based on the G–R parameter was also found that the addition of CR decreased fatigue resistance, while WMA improved it. Iterlow T had a more noticeable effect than Evotherm M1.
- From the MSCR test, with the addition of CR, lower *J_nr_* values were obtained; these *J_nr_* values showed a slight increase with the addition of WMA. On the other hand, the recovery percentage of asphalt binder increased with CR, and with the addition of WMA, these percentages decreased. Likewise, the Superpave and Shenoy parameters correlated with *J_nr_* in the rutting response.

Considering the above-mentioned conclusions, using WMA for modified asphalt binders with CR can mean a decrease in the environmental damage caused. This study can help to establish a mixing protocol and to base regulation on this type of modification for asphalt binders. WMAs are recommended for content higher than 15% because with this CR content, WMAs do not provide good performance.

Considering the limitations and the experimental methodology employed, the results obtained in this research cannot be generalised. In addition, the following topics are recommended for future research: (i) analysis of different asphalt binders, and (ii) long-term ageing study and analysis of chemical properties, and the assessment of ageing and chemical properties through Fourier transform infrared spectroscopy (FTIR), and SARA fractions. Likewise, for a better understanding of the fatigue behaviour of asphalt binders, it is suggested to perform the linear amplitude sweep (LAS) test.

## Figures and Tables

**Table 1:** Properties of the asphalt binder used in this research.

Characteristic	Units	ASTM Standard	Results
Penetration (25 °C, 100 g, 5 s)	0.1 mm	ASTM D5	60.7
Softening point	°C	ASTM D36	49
Penetration index	-	ASTM D5	−1.0
Absolute viscosity (60 °C)	P	ASTM D2171	2290
Ductility at 25 °C	cm	ASTM D113	>100
Trichloroethylene solubility	%	ASTM D2042	99.99
Water content	%	ASTM D2216	0.0
Ignition point through open Cleveland cup	°C	ASTM D92	286
Kerosene content	%	ASTM D187	1.3
Specific gravity 25 °C	kg/m^3^	ASTM D70	1030

**Table 2:** Physical properties of the chemical-based additives used in this study.

Property	Evotherm M1	Iterlow T
Colour	Dark Amber	Yellow-dark amber
Physical state	Liquid	Liquid
Density at 25 °C (g/cm^3^)	0.97	0.95–1.05
Viscosity at 25 °C (cP)	130–280	150–250

**Table 3:** Optimum mixing time for different percentages of CR.

CR Percentage (%)	Optimum Mixing Time (min)
15	45
18	45
21	60

**Table 4:** The effect of CR content, and WMA additives on the values of SHRP. The ratio of rutting parameter of i% CR-modified binder to that of modified with j% CR.

i and j Indices	Unaged		RTFO	
	$\frac{G^{*}/\sin\delta_{i}}{G^{*}/\sin\delta_{j}}$	$\frac{G^{*}/(1-{\left(1/\left(tg\delta \sin\delta \right)\right)}_{i}}{G^{*}/(1-{\left(1/\left(tg\delta \sin\delta \right)\right)}_{j}}$	$\frac{G*/\sin\delta_{i}}{G*/\sin\delta_{j}}$	$\frac{G^{*}/(1-{\left(1/\left(tg\delta \sin\delta \right)\right)}_{i}}{G^{*}/(1-{\left(1/\left(tg\delta \sin\delta \right)\right)}_{j}}$
CR-Modified Binders				
i = 15, j = 0	4.22	5.35	7.15	14.65
i = 18, j = 0	5.27	6.73	11.07	21.91
i = 21, j = 0	8.62	14.14	13.18	29.78
i = 18, j = 15	1.25	1.26	1.55	1.50
i = 21, j = 15	2.04	2.64	1.84	2.03
i = 21, j = 18	1.64	2.10	1.19	1.36
CR + Evotherm M1				
i = 15, j = 0	0.76	6.19	5.83	11.50
i = 18, j = 0	1.11	9.41	7.44	13.77
i = 21, j = 0	1.65	15.86	12.61	27.48
i = 18, j = 15	1.45	1.52	1.28	1.19
i = 21, j = 15	2.18	2.56	2.16	2.39
i = 21, j = 18	1.49	1.69	1.70	1.99
CR + Iterlow T				
i = 15, j = 0	4.77	5.94	4.82	9.80
i = 18, j = 0	6.16	7.88	9.43	18.04
i = 21, j = 0	11.14	19.23	15.7	35.83
i = 18, j = 15	1.29	1.33	1.96	1.84
i = 21, j = 15	2.34	3.24	3.27	3.66
i = 21, j = 18	1.81	2.44	1.67	1.99

**Table 5:** Results of MSCR test.

Asphalt Binders	*J_nr_*		R	Traffic Level
	*J_nr_*_3.2_ (kPa^−1^)	*J_nrslope_* (%)	R_3.2_ (%)	
CONTROL	3.97	12.77	13.83	S
0.3 EVO	4.06	2.58	11.54	S
0.3 ITERLOW	4.14	13.45	11.54	S
15% CR	0.26	4.73	60.85	E
18% CR	0.216	3.96	61.63	E
21% CR	0.143	2.49	83.52	E
15% CR + EVO	0.44	8.16	25.83	E
18% CR + EVO	0.31	4.39	58.54	E
21% CR + EVO	0.23	3.39	77.84	E
15% CR + ITER	0.394	8.32	31.34	E
18% CR + ITER	0.338	7.26	53.83	E
21% CR + ITER	0.217	3.581	74.73	E

**Table 6:** Statistical analysis of rheological tests.

Asphalt Binders	Viscosity at 135 °C (cP)	Viscosity at 175 °C (cP)	G*/sin *δ* at 64 °C (kPa)	*J_nr_* at 64 °C (kPa^−1^)	G–R (kPa)
Effect of EVO modification					
0.3 EVO vs. Control	No	No	Y (↓)	No	No
15 CR + EVO vs. 15 CR	No	No	Y (↓)	Y (↑)	No
18 CR + EVO vs. 18 CR	Y (↓)	Y (↓)	Y (↓)	Y (↑)	No
21 CR + EVO vs. 21 CR	No	No	Y (↓)	Y (↑)	No
Effect of ITER modification					
0.3 ITERLOW vs. Control	No	No	Y (↓)	Y (↑)	Y (↓)
15 CR + ITER vs. 15 CR	No	No	Y (↓)	No	No
18 CR + ITER vs. 18 CR	Y (↓)	No	Y (↓)	Y (↑)	No
21 CR + ITER vs. 21 CR	No	No	Y (↓)	Y (↑)	No
Effect of CR modification					
Control vs. 15 CR	Y (↑)	Y (↑)	Y (↑)	Y (↓)	Y (↑)
Control vs. 18 CR	Y (↑)	Y (↑)	Y (↑)	Y (↓)	Y (↑)
Control vs. 21 CR	Y (↑)	Y (↑)	Y (↑)	Y (↓)	Y (↑)
15 CR vs. 18 CR	Y (↑)	Y (↑)	Y (↑)	No	Y (↑)
15 CR vs. 21 CR	Y (↑)	Y (↑)	Y (↑)	Y (↓)	Y (↑)
18 CR vs. 21 CR	No	Y (↑)	Y (↑)	No	Y (↑)
0.3 EVO vs. 15 CR + EVO	Y (↑)	Y (↑)	Y (↑)	Y (↓)	Y (↑)
0.3 EVO vs. 18 CR + EVO	Y (↑)	Y (↑)	Y (↑)	Y (↓)	Y (↑)
0.3 EVO vs. 21 CR + EVO	Y (↑)	Y (↑)	Y (↑)	Y (↓)	Y (↑)
15 CR + EVO vs. 18 CR + EVO	Y (↑)	Y (↑)	Y (↑)	Y (↓)	Y (↑)
15 CR + EVO vs. 21 CR + EVO	Y (↑)	Y (↑)	Y (↑)	Y (↓)	Y (↑)
18 CR + EVO vs. 21 CR + EVO	Y (↑)	Y (↑)	Y (↑)	No	Y (↑)
0.3 ITERLOW vs. 15CR+ITER	Y (↑)	Y (↑)	Y (↑)	Y (↓)	Y (↑)
0.3 ITERLOW vs. 18 CR + ITER	Y (↑)	Y (↑)	Y (↑)	Y (↓)	Y (↑)
0.3 ITERLOW vs. 21 CR + ITER	Y (↑)	Y (↑)	Y (↑)	Y (↓)	Y (↑)
15 CR + ITER vs. 18 CR + ITER	Y (↑)	Y (↑)	Y (↑)	No	Y (↑)
15 C + ITER vs. 21 CR + ITER	Y (↑)	Y (↑)	Y (↑)	Y (↓)	Y (↑)
18 CR + ITER vs. 2 1CR + ITER	Y (↑)	Y (↑)	Y (↑)	Y (↓)	Y (↑)

Note: Y (↑) and Y (↓) indicates significantly increment and decrement in value, respectively.

## Data Availability

Data are available upon request to the corresponding author.

## Abbreviations

AASHTO	American Association of State Highway and Transportation Officials
ASTM	American Society for Testing and Materials
CR	Crumb Rubber
DSR	Dynamic Shear Rheometer
EVO	Evotherm M1
FTIR	Fourier Transform Infrared Spectroscopy
*G**	Complex Modulus
G–R	Glover–Rowe
HMA	Hot Mix Asphalt
IDU	Institute of Urban Development
ITER	Iterlow T
*J_nr_*	Non-Recoverable Creep Compliance
*J_nrdiff_*	Non-Recoverable Creep Compliance Differential
*J_nrslope_*	Non-Recoverable Creep Compliance Slope
LAS	Linear Amplitude Sweep
MSCR	Multiple Stress Creep Recovery
PG	Performance Grade
PMB	Polymer-Modified Binders
R	Percentage Recovery
RAP	Reclaimed Asphalt Pavement
RTFO	Rolling Thin Film Oven
RV	Rotational Viscosity
SBR	Styrene-Butadiene-Rubber
SBS	Styrene-Butadiene-Styrene
SEM	Scanning Electron Microscopy
SHRP	Strategic Highway Research Program
WMA	Warm Mix Asphalt
*δ*	Phase Angle
